# Novel Nutrition-Based Nomograms to Assess the Outcomes of Lung Cancer Patients Treated With Anlotinib or Apatinib

**DOI:** 10.3389/fonc.2021.628693

**Published:** 2021-03-08

**Authors:** Hui Zheng, Qin Pan, Wenchao Zhu, Hongsen Li, Zhongfeng Niu, Yong Fang, Da Li, Haizhou Lou, Hong Hu, Jiawei Shou, Hongming Pan

**Affiliations:** ^1^ Department of Medical Oncology, Sir Run Run Shaw Hospital, College of Medicine, Zhejiang University, Hangzhou, China; ^2^ Department of Radiology, Sir Run Run Shaw Hospital, College of Medicine, Zhejiang University, Hangzhou, China

**Keywords:** body composition, weight, antiangiogenic, lung cancer, nomogram

## Abstract

**Background:**

Previous studies have indicated that the changes in body composition during treatment are prognostic in lung cancer. The question which follows is it may be too late to identify vulnerable patients after treatment and to improve outcomes for these patients. In our study, we sought to explore the alterations of body composition and weight before the outset of the antiangiogenic treatment and its role in predicting clinical response and outcomes.

**Methods:**

In this retrospective study, 122 patients with advanced lung cancer treated with anlotinib or apatinib were analyzed. The changes in weight and body composition including skeletal muscle index (SMI), subcutaneous adipose tissue (SAT), and visceral adipose tissue (VAT) for 3 months before the outset of antiangiogenic treatment and other clinical characteristics were evaluated with LASSO Cox regression and multivariate Cox regression analysis, which were applied to construct nomograms. The performance of the nomograms was validated internally by using bootstrap method with 1,000 resamples models and was assessed by the concordance index (C-index), calibration plots, decision curve analysis (DCA).

**Results:**

The median progression-free survival (PFS) and overall survival (OS) were 128 (95% CI 103.2–152.8) days and 292 (95% CI 270.9–313.1) days. Eastern Cooperative Oncology Group performance status (ECOG PS), brain metastases, the Glasgow Prognostic Score (GPS), clinical response, therapeutic regimen, and ΔL1SMI per 90 days were significantly associated with PFS, while ECOG PS, GPS, clinical response, therapeutic regimen, ΔL1SMI per 90 days were identified for OS. The C-index for the nomograms of PFS and OS were 0.763 and 0.748, respectively. The calibration curves indicated excellent agreement between the predicted and actual survival outcomes of 3- and 4-month PFS and 7- and 8-month OS. DCA showed the considerable value of the model.

**Conclusion:**

Nomograms were developed from clinical features and nutritional indicators to predict the probability of achieving 3-month and 4-month PFS and 7-month and 8-month OS with antiangiogenic therapy for advanced lung cancer. Dynamic changes in body composition before the initiation of treatment contributed to early detection of poor outcome.

## Introduction

Cancer cachexia was characterized by the ongoing loss of skeletal muscle mass (with or without the loss of fat mass) in 2011 (1). Numerous studies have indicated that low muscle mass at baseline is an independent predictor of survival in lung cancer (2–5). In more recent studies, longitudinal skeletal muscle alteration but not low muscle mass at baseline was recognized as a significant prognostic factor in lung cancer and other types of cancer (6–8). The loss of fat mass also represents an important property of cachexia. Some studies even suggested that whole body fat predicted survival, whereas lean tissue mass did not, and body fat loss preceded lean tissue loss in gastrointestinal tumors (9). A pilot study of pancreatic cancer found that higher visceral adipose tissue loss (and age) predicted poorer survival in multivariate analyses (7). The predictive value of body composition alterations for prognosis has also been studied in patients receiving multikinase inhibitors with antiangiogenic action. Kenji et al. reported that the rapid depletion of subcutaneous fat mass and skeletal muscle mass (SMM) were effective markers for the outcomes of patients with hepatocellular carcinoma treated with sorafenib (10). Hiroki et al. suggested that decreased SMM indicated poor prognosis in patients undergoing first-line sunitinib therapy for metastatic renal cell carcinoma (11). Although the changes in body composition during treatment have been confirmed to be prognostic according to the above research, the question which follows is it may be too late to identify vulnerable patients after treatment and to improve outcomes for these patients. In our study, we sought to explore the alterations of body composition and weight before the outset of the target treatment and their roles in predicting clinical response and outcomes.

Lung cancer is by far the most common cancer with the highest incidence rate and mortality rate in the world and in China (12). Although the treatment of lung cancer has made considerable progression in recent years, there are limited treatment options for patients experiencing progression after two or more lines of standard treatment. Novel small-molecule TKIs that inhibit tumor angiogenesis have emerged as potential options. Anlotinib inhibits vascular endothelial growth factor receptor (VEGFR) types 2 and 3, fibroblast growth factor receptor (FGFR) types 1–4, platelet-derived growth factor receptor (PDGFR) types *α* and *β*, stem cell factor receptor(c-Kit), and Ret (13). After the results of ALTER-0303 were published (14), anlotinib (AL3818) hydrochloride has been recognized as a third-line treatment option for refractory advanced NSCLC since 2018. In addition, a phase II study of ALTER1202 demonstrated that anlotinib provides a progression-free survival benefit for SCLC (15). Apatinib (HengRui Pharmaceutical Co, Ltd, Lianyungang, People’s Republic of China), which selectively inhibits VEGFR-2, is a TKI with indicated efficacy in chemotherapy-refractory lung cancer patients (16–18). Nutritional issues may be overlooked by physicians during antiangiogenic treatment, for the reason that fatigue and digestive adverse events such as anorexia, nausea, and vomiting, which cause malnutrition and unfavorable changes in body composition, are less common in the treatment of anlotinib or apatinib compared with chemotherapy. The concern with dynamic changes in body composition before the initiation of targeted therapy may be helpful to identify patients with nutritional issues early on. Therefore, it is important to construct a prognostic model to guide the selection of treatment based on pre-treatment clinical and nutritional characteristics.

Computed tomography (CT) is universally used to monitor disease progression and evaluate clinical response for treatment in lung cancer and can provide information about body composition without additional costs and radiation exposure. CT analysis dependent on a slice at the third lumbar vertebra level (L3) is now well established for measuring muscle quality and quantity (19), whereas chest scans generally do not reach beyond the first lumbar level (L1). The pectoralis muscle (adjusted model R^2^ = 0.76) has been proposed as an alternative in COPD research (20, 21). Karin et al. reported that L1 (r = 0.90, *P* < 0.001) is a better alternative than the pectoralis muscle (r = 0.71, *P* < 0.001) to substitute L3 for muscle measurement in lung cancer (8). Shen et al. indicated an excellent correlation at the L1 level (r = 0.903, *P* < 0.001) for skeletal muscle mass measurement (22), and the L1 level is covered in most chest CTs.

In this study, we aim to examine alterations of body composition and weight for 3 months before the outset of antiangiogenic treatment and establish nomograms based on the above nutritional indicators and other clinical characteristics to individually predict long-term outcomes in lung cancer patients receiving antiangiogenic treatment of anlotinib or apatinib.

## Materials and Methods

### Patients and Study Design

This retrospective study was based on a review of electronic medical records from patients pathologically diagnosed with lung cancer and treated with anlotinib or apatinib between October 2016 and October 2019 at Sir Run Run Shaw Hospital. The primary inclusion criteria were as follows: stage IV lung cancer, age ≥18 years, expected survival time >3 months before the treatment of anlotinib or apatinib, complete records of laboratory reports, and chest CTs from three months before baseline to the time of disease progression. Enrolees could be receiving targeted therapy with anlotinib or apatinib alone or in combination with chemotherapy, immunotherapy, or EGFR-targeting agents at the doctors’ discretion. Patients were excluded if they received radiotherapy or interventional therapy during antiangiogenic treatment. The relative changes in weight and body composition (SMI: skeletal muscle index, VAT: visceral adipose tissue, and SAT: subcutaneous adipose tissue measured at the L1 level) per 90 days before the initiation of antiangiogenic treatment and other clinical characteristics were explored to predict prognosis.

This study conformed to the Declaration of Helsinki and also was approved by the institutional review board of Sir Run Run Shaw Hospital. All patients had signed informed consent.

### Radiological Evaluation

The chest CT scans performed within two weeks and within three months before treatment with anlotinib or apatinib were collected as baseline scans and pre-treatment scans, respectively. The CT scans performed at the discretion of the doctors for response assessment were also collected. In every collected chest CT scan, the skeletal muscle, visceral adipose tissue, and subcutaneous adipose tissue at the L1 level with both vertebral transverse processes visible were used in the analysis (8, 23, 24).

The cross-sectional areas of the muscle (cm^2^) at the L1 level computed from each slice were normalized by the square of the height (m^2^) to obtain the L1SMI (cm^2^/m^2^). To assess changes in body composition in different patients, the change between the baseline scans and pre-treatment scans was divided by the interval and then multiplied by 90 to obtain the change per 90 days before the initiation of antiangiogenic treatment.

CT acquisition parameters were as follows: non-enhanced, slice thickness was 5 mm, and tube voltage was 120 kV. Quantitative measurements were performed by a trained radiologist (Zhu) using Slice O’ Matic v 5.0 software (Tomovision, Canada). Established thresholds in Hounsfield units were as follows: skeletal muscle −29 to 150, SAT −190 to −30, and VAT −150 to −50. Boundaries were defined artificially by drawing regions of interests using established cut-off thresholds.

### Other Assessments

Clinical data, including demographics, tumor stage, treatment information, Eastern Cooperative Oncology Group performance status (ECOG PS), smoking history, blood counts, and biochemical tests at baseline, were collected from electronic medical records. Body mass index (BMI) was calculated as the weight in kilograms divided by the height in meters squared. The Glasgow Prognostic Score (GPS) was recorded according to C-reactive protein (CRP) and albumin (GPS = 0: albumin >35g/L and CRP <10 mg/L; GPS = 1: albumin <35 g/L or CRP >10 mg/L; GPS = 2: albumin<35 g/L and CRP >10 mg/L) (25). The clinical response was assessed according to the Response Evaluation Criteria in Solid Tumors (RECIST), version 1.1. We confirmed the survival status and the date of death by follow-up until October 1^st^, 2020. Progression-free survival (PFS) was defined as the time from the start of treatment with anlotinib or apatinib to disease progression or death. Overall survival (OS) was defined as the time from the start of treatment with anlotinib or apatinib to death resulting from any cause.

### Statistical Analysis

The statistical analysis was performed using R (version 4.0.3). Patient characteristics were compared using Student’s *t*-test for normally distributed continuous variables, the Mann−Whitney U test for non-normally distributed continuous variables, and the χ^2^ test for categorical variables. The two-sided *P* value <0.05 was considered statistically significant. The Kaplan−Meier method was used to estimate survival, and differences were compared by the log-rank test. According to the overall survival status, the optimal cut-off values for body composition alterations (L1SMI, L1VAT, and L1SAT) were defined as the point that gave the most significant log-rank cohort split.

The least absolute shrinkage and selection operator (LASSO) method was used to primarily select potential predictive features to solve the collinearity and avoid over-fitting to some extent. Selected predictive factors were further included in the multivariate analysis using a Cox proportional hazards model. Based on identified predictive factors for PFS and OS in the final model, nomograms to predict the probability of disease progression at 3 and 4 months and death probability at 7 and 8 months for lung cancer patients treated with anlotinib or apatinib were constructed and then validated internally by using bootstrap method with 1,000 resamples. The value of Concordance index (C-index) ranging from 0.5 to 1.0 was used to evaluate the discriminative abilities of the nomograms. Calibration curves (1,000 bootstrap resamples) were applied to test the consistency between the predicted and actual 3- and 4- month PFS, and 7- and 8- month OS. Decision curve analysis (DCA) was generated to evaluate the latent value of the prediction model.

## Results

### Patient Characteristics

A total of 148 consecutive patients were enrolled in the study. Of these, 122 patients met the inclusion criteria. The reasons for exclusion were rapid disease progression (n = 5), unavailable chest CT scans before or after treatment with anlotinib or apatinib (n = 6), and unacceptable quality (such as artifact and unavailability of L1 level) for L1 evaluation (n = 15). Among 122 patients, 32 patients (26.2%) received combined therapy. For combined chemotherapy, paclitaxel (n = 1) or docetaxel (n = 6) was used in combination with anlotinib, and docetaxel (n = 2) was used in combination with apatinib. For combined immunotherapy, PD1 monoclonal antibodies were administered with anlotinib (n = 10) or apatinib (n = 2). For combined targeted therapy, first-generation EGFR TKIs were prescribed with anlotinib (n = 1) or apatinib (n = 10). All patient characteristics are shown in **Table 1**. 96(78.6%) were men, and 26(21.3%) were women. Their median age was 62.5 years.

**Table 1:** Patient characteristics.

Characteristics	All (n = 122)	Anlotinib (n = 71)	Apatinib (n = 51)	*P*
Age(years)^*^	62.5(±8.8)	63.4(±9.3)	61.4(±8.0)	0.204
Sex(male/female,n)	96/26	52/19	44/7	0.083*^c^*
ECOG PS(n)				0.198*^c^*
0–1	96(78.7%)	53(74.6%)	43(84.3%)	
2–3	26(21.3%)	18(25.4%)	8(15.7%)	
Smoking status(n)				0.541*^c^*
Never smoker	59(48.4%)	36(50.7%)	23(45.1%)	
Current or former smoker	63(51.6%)	35(49.3%)	28(54.9%)	
Pathological classification(n)				0.111*^c^*
adenocarcinoma	62(50.8%)	41(57.7%)	21(41.2%)	
squamous	36(29.5%)	20(28.2%)	14(27.5%)	
small cell	24(19.7%)	10(14.1%)	16(31.4%)	
Prior treatment line^#^	2.28(0–6)	2.4(0–6)	2.1(1–5)	0.154*^a^*
Combination therapy(n)	32(26.2%)	18(25.4%)	14(27.5%)	<0.001*^b^*
combined chemotherapy	9(7.4%)	7(9.9%)	2(3.9%)	
combined immunotherapy	12(9.8%)	10(14.1%)	2(3.9%)	
combined target therapy	11(9.0%)	1(1.4%)	10(19.6%)	
Brain metastases(n)	24(19.7%)	18(25.4%)	6(11.8%)	0.063*^c^*
Therapeutic effect(PR + SD/PD)	95/27	53/18	42/9	0.312*^c^*
Baseline GPS(0/1/2,n)	58/39/25	28/23/20	30/16/5	0.027*^c^*
Body mass index (kg/m^2^)^*^	22.7(±3.0)	22.7(±3.3)	22.6(±2.7)	0.853
ΔL1SMI(cm^2^/m^2^/90 days)^*^	−2.9(±5.2)	−2.6(±6.1)	−3.3(±3.8)	0.436
ΔL1VAT(cm^2^/90 days)^*^	−4.5(±40.5)	−1.2(±43.3)	−9.0(±36.3)	0.298
ΔL1SAT(cm^2^/90 days)^*^	−-3.7(±20.0)	−3.9(±23.7)	−3.4(±13.6)	0.883
Δweight(kg/90 days)^*^	−0.7(±4.5)	−0.7(±5.2)	−0.5(±3.3)	0.809

*mean ± standard deviation presented; ^#^mean (range) ^a^Mann–Whitney U test ^b^Fisher’s exact test ^c^the χ2 test; ECOG PS, Eastern Cooperative Oncology Group performance status; GPS, Glasgow Prognostic Score; PD, progressive disease; PR, partial response; SD, stable disease; L1SMI, skeletal muscle index at the first lumbar vertebra level; L1VAT, visceral adipose tissue at the first lumbar vertebra level; L1SAT, subcutaneous adipose tissue at the first lumbar vertebra level.

### The Relationship Between Alterations of Weight and Body Composition and Clinical Response

**Figure 1:**
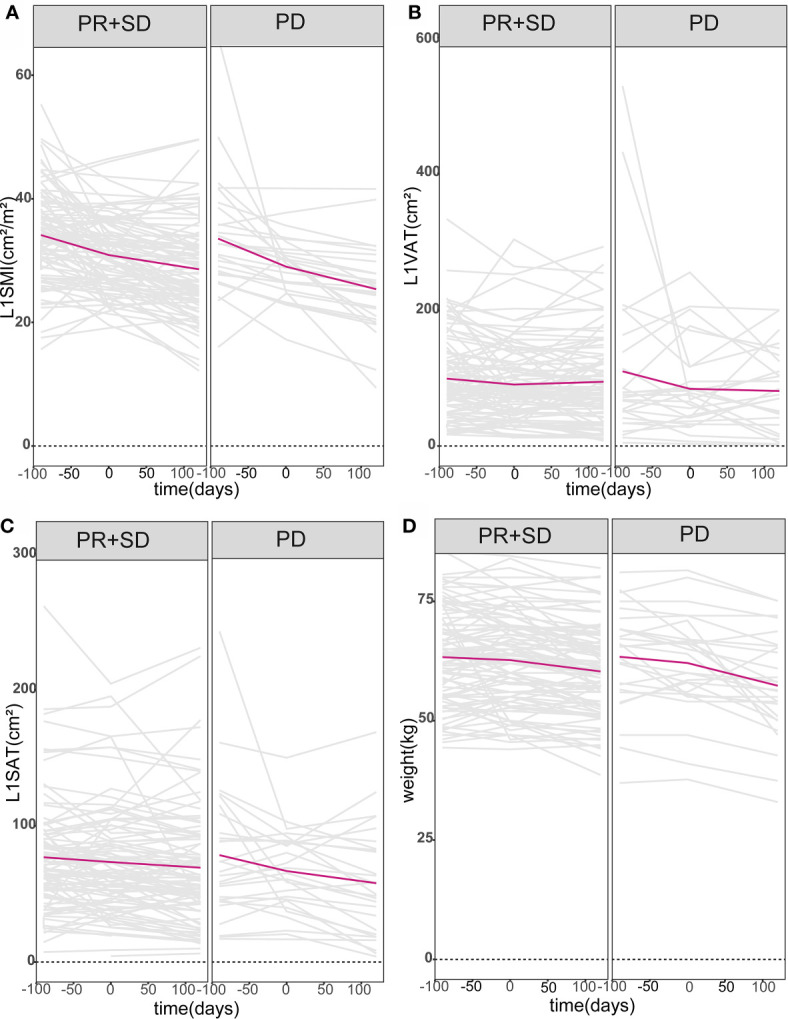
Body composition and weight trajectories over time. Patient-specific longitudinal trajectories of L1SMI, L1VAT, L1SAT, and weight over time were depicted in **(A–D)**. All patients were divided into two groups according to whether they had PD or not. Lines in gray represent the individual patient body composition and weight trajectories, while bold lines in red denote the mean populations of body composition and weight trajectories over time. L1SMI, skeletal muscle index at the first lumbar vertebra level; L1VAT, visceral adipose tissue at the first lumbar vertebra level; L1SAT, subcutaneous adipose tissue at the first lumbar vertebra level.

The objective response rate was 8.2% (n = 10), all based on achieving PR. Moreover, 69.7% of the patients had SD, 22.1% had PD, and the disease control rate was 77.9%. The fold line diagram was performed to explore the relationship between alterations of weight and body composition and clinical response. **Figure 1** shows the weight and body composition trajectories before and after the initiation of anlotinib or apatinib. The time scales were adjusted to each patient’s time to the baseline CT scan. Decrease in weight and body composition over time were detected in all patients, but appeared more pronounced in patients who had PD, compared to patients achieving PR or SD. For patients with unfavorable efficacy of antiangiogenic treatment, early prediction of efficacy may be possible since steeper slopes in alterations of weight and body composition had emerged before treatment in these patients.

### Construction and Validation of the Prognostic Nomograms for PFS and OS

Over a median follow-up duration of 259.7 (range 61−1,124) days, of 122 patients, 107 patients experienced disease progression, and 63 patients died. The median PFS and OS were 128 (95% CI 103.2−152.8) days and 292 (95% CI 270.9−313.1) days, respectively. The 3- and 4-month PFS rate was 68.3 and 53.5% for all patients. The 7- and 8-month OS rate was 74.3 and 65.6, respectively. We conducted PFS and OS analysis of all patients stratified by clinical characteristics and alterations of body composition and weight per 90 days before the baseline (**Table 2**). The changes in L1SMI, L1VAT, and L1SAT per 90 days before the baseline were all significantly associated with PFS and OS according to Kaplan−Meier analysis.

**Table 2:** Kaplan–Meier analysis for progression-free survival and overall survival stratified by clinical factors.

Factors	PFS		OS	
	Median, 95% CI (days)	*P*	Median, 95% CI (days)	*P*
Age, years		0.241		0.476
<55	124 (40.9–207.1)		299 (253.3–344.7)	
55–70	134 (105.6–162.4)		273 (233.0–313.0)	
>70	128 (103.2–152.8)		293 (146.4–439.7)	
Drug		0.353		0.064
anlotinib	117 (94.3–139.7)		316 (245.9–386.1)	
apatinib	138 (103.9–172.1)		238 (175.2–300.8)	
Sex		0.601		0.666
female	126 (95.1–156.9)		293 (254.9–331.1)	
male	128 (97.7–158.3)		290 (249.0–331.0)	
ECOG PS		**<0.001**		**0.002**
0–1	151 (132.4–169.6)		316 (259.5–372.5)	
2–3	75 (70.0-80.0)		211 (122.4–299.6)	
Smoking status		0.127		0.164
Never smoker	149 (123.5–174.5)		299 (232.8–365.2)	
Current or former smoker	111 (78.3–143.7)		284 (235.8–332.2)	
Pathological classification		0.949		0.045
adenocarcinoma	113 (81.0–145.0)		380 (147.8–612.2)	
squamous	144 (120.9–167.1)		282 (246.8–317.2)	
small cell	135 (105.0–165.0)		238 (176.8–299.2)	
Prior treatment line		0.824		0.286
<3	124 (93.2–154.8)		316 (207.9–424.1)	
≥3	133 (96.0–170.0)		284 (240.4–327.6)	
Brain metastases		0.147		0.932
No	`134 (103.5–164.5)		292 (265.3–318.7)	
Yes	113 (52.5–173.5)		293 (233.4–352.6)	
GPS		**<0.001**		**0.001**
0	167 (137.0–197.0)		299 (188.3–409.7)	
1	113 (82.4–143.6)		299 (214.7–383.3)	
2	77 (68.8–85.2)		175 (159.2–190.8)	
PD *vs* PR/SD		**<0.001**		**<0.001**
PD	52 (40.1–63.9)		178 (133.0–223.0)	
PR/SD	149 (128.8–169.2)		316 (258.8–373.2)	
Therapeutic regimen		**0.004**		**<0.001**
Single drug	111 (86.9–135.1)		256 (191.1–320.9)	
Combined chemo	149 (143.2–154.8)		–	
Combined immune	186 (46.4–325.6)		403 (185.2–620.8)	
Combined target	231 (116.6–345.4)		–	
BMI		0.211		0.165
<18.5	113 (68.2–157.8)		218 (149.7–286.3)	
18.5–25	133 (97.8–168.2)		296 (251.1–340.9)	
>25	126 (43.7–208.3)		238 (203.1–272.9)	
ΔL1SMI per 90 days^*^		**0.042**		**0.002**
≤−3.96 (cm^2^/m^2^)	124 (78.4–169.6)		254 (187.0–321.0)	
>−3.96 (cm^2^/m^2^)	134 (101.2–166.8)		380 (263.0–497.0)	
ΔL1VAT per 90 days^*^		**0.004**		**0.003**
≤4.68 (cm^2^)	113 (83.3–141.7)		277 (247.0-307.0)	
>4.68 (cm^2^)	162 (138.3–185.7)		NA	
ΔL1SAT per 90 days^*^		**0.042**		**0.003**
≤ −8.25 (cm^2^)	149 (99.5–198.5)		256 (197.3–314.7)	
>−8.25 (cm^2^)	117 (83.8–150.2)		NA	
Δweight per 90 days^*^		**0.096**		0.107
≤ −2.28 (kg)	117(99.5–134.5)		270 (216.6–323.4)	
>−2.28 (kg)	137 (99.8–174.2)		299 (210.7–387.3)	

ECOG PS, Eastern Cooperative Oncology Group performance status; GPS, Glasgow Prognostic Score; PD, progressive disease; PR, partial response; SD, stable disease; BMI, body mass index; L1SMI, skeletal muscle index at the first lumbar vertebra level; L1VAT, visceral adipose tissue at the first lumbar vertebra level; L1SAT, subcutaneous adipose tissue at the first lumbar vertebra level;

*According to the overall survival status, the optimal cut-off values for body composition alterations (ΔL1SMI, Δ L1VAT, and ΔL1SAT) and Δweight were defined as the point that gave the most significant log-rank cohort split.

The bold value means P<0.05.

**Figure 2:**
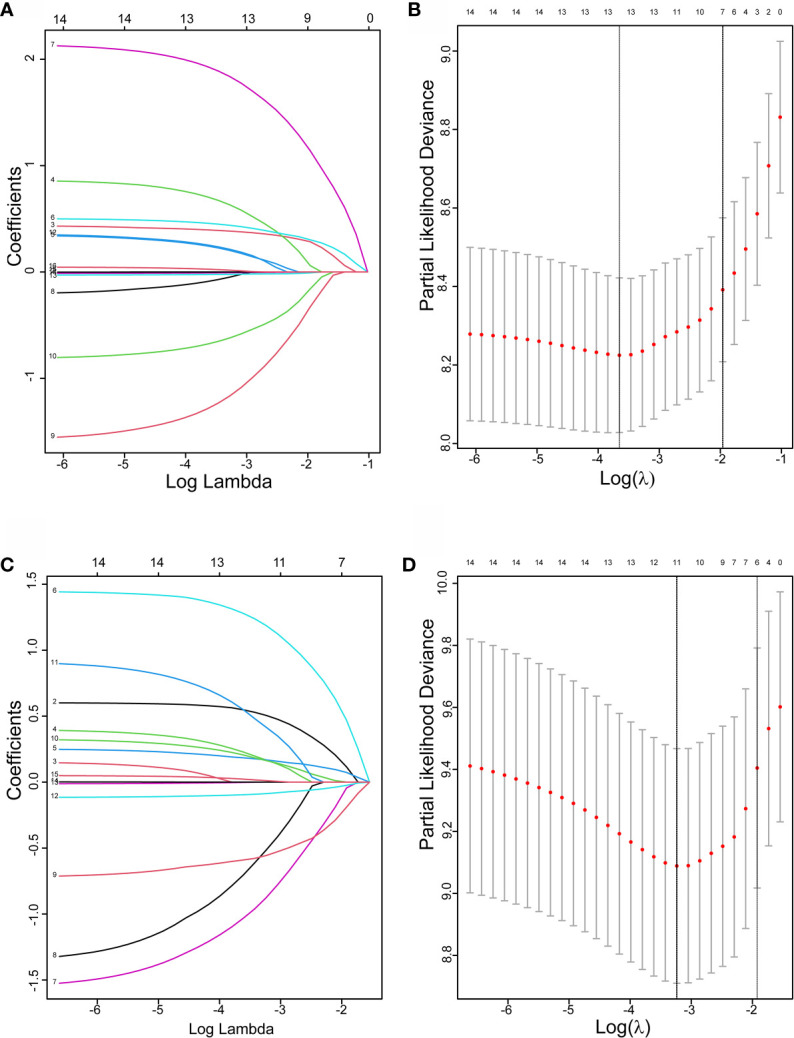
The LASSO regression used to select prognostic factors. **(A)** LASSO coefficient profiles of 13 variables for PFS; **(B)** LASSO Cox analysis identified six variables for PFS; **(C)** LASSO coefficient profiles of 13 variables for OS; **(D)** Lasso Cox analysis identified six variables for OS; PFS, progression free survival; OS, overall survival.

**Figure 3:**
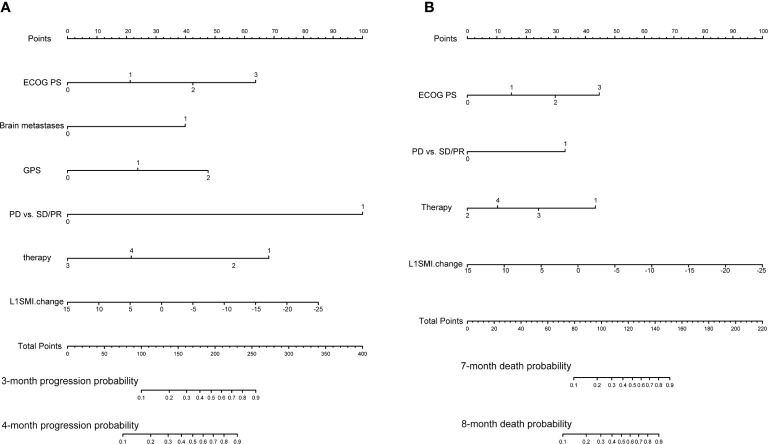
Predictive nomograms. **(A)** Nomogram for predicting 3- and 4-month probability of disease progression **(B)** Nomogram for predicting 7- and 8-month death probability. ECOG PS, Eastern Cooperative Oncology Group performance status; GPS, Glasgow Prognostic Score; PD, progressive disease; PR, partial response; SD stable disease; L1SMI, skeletal muscle index at the first lumbar vertebra level.

**Figure 4:**
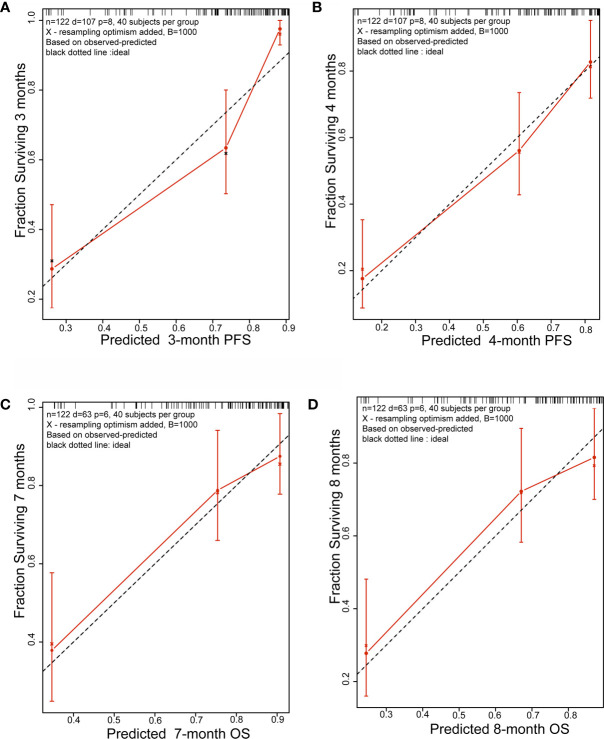
Calibration plots. **(A)** 3-month and **(B)** 4-month PFS nomogram calibration plots; **(C)** 7-month and **(D)** 8-month OS nomogram calibration plots; PFS, progression free survival; OS, overall survival.

**Figure 5:**
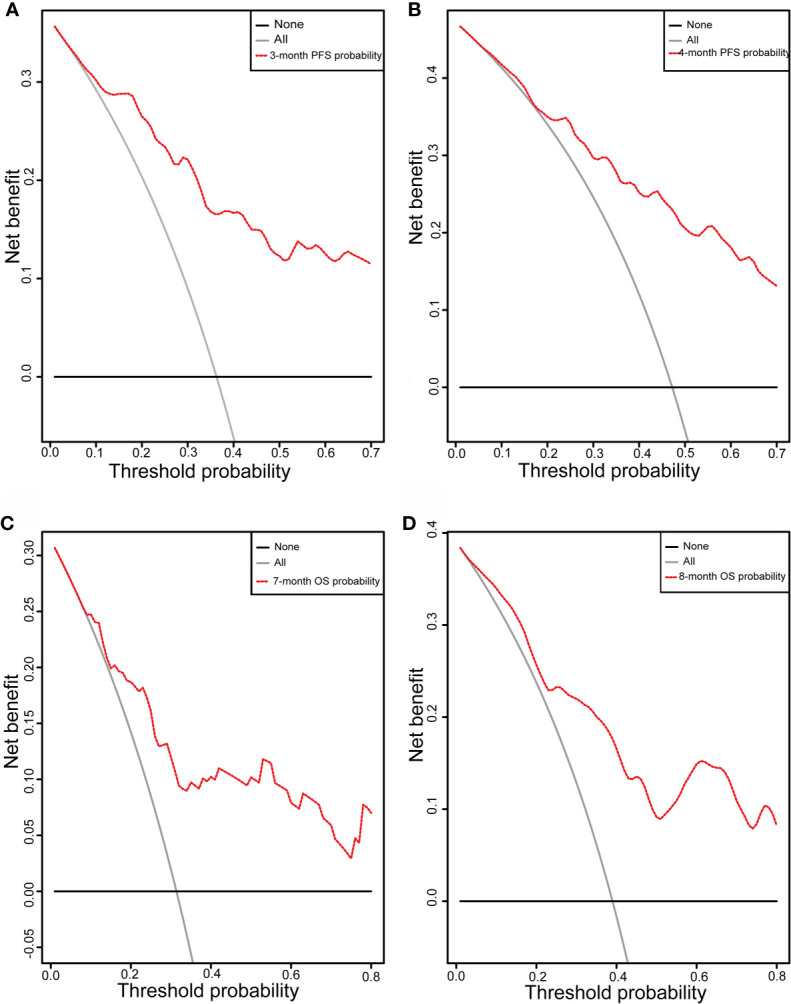
DCA curve. DCA curve of the nomograms for **(A)** 3-month and **(B)** 4-month PFS and **(C)** 7-month and **(D)** 8-month OS; X-axis represents threshold probabilities, and Y-axis measures net benefit. The horizontal line along X-axis assumes that disease progression or overall death occurred in no patients, whereas solid gray line assumes that all patients will have disease progression or overall death at a specific threshold probability. Red dashed line represents nomogram. PFS, progression free survival; OS, overall survival.

Initially, 13 variables were included in the analysis. Based on the results of LASSO Cox regression analysis, ECOG PS, brain metastases, GPS, PD *vs* PR/SD, therapeutic regimen, and ΔL1SMI per 90 days were screened out for PFS, while ECOG PS, GPS, PD *vs* PR/SD, therapeutic regimen, prior treatment line, and ΔL1SMI per 90 days were identified for OS (**Figure 2**). In the multivariate analysis of above selected factors, six factors were all independently and significantly associated with PFS, while ECOG PS, PD *vs* PR/SD, therapeutic regimen, ΔL1SMI per 90 days were independently and significantly associated with OS (**Table 3**). Based on the above, we developed two nomograms to predict PFS and OS (**Figure 3**). The first was developed to predict the probability of progression disease at 3 and 4 months after treatment based on six factors including ECOG PS, brain metastases, GPS, PD *vs* PR/SD, therapeutic regimen, and ΔL1SMI per 90 days. The second was established to predict death probability at 7 and 8 months based on ECOG PS, PD *vs* PR/SD, therapeutic regimen, and ΔL1SMI per 90 days. The C-indexes for the nomograms of PFS and OS were 0.763 and 0.748, respectively. The similarities between the actual observation and predicted survival rates of nomograms were validated by plotting a calibration curve of PFS and OS. The x-axis represents the predicted probability estimated by the nomograms, and the observed events is shown on the y-axis. The calibration curves indicated excellent agreement between the predicted and actual survival outcomes of 3- and 4-month PFS and 7- and 8-month OS (**Figure 4**). DCA showed the considerable value of the model and the novel nomograms showed net benefit across the range of decision threshold probabilities (**Figure 5**).

**Table 3:** Multivariate Cox analysis of the training cohort based on the results of lasso regression.

Factors	PFS		OS	
	HR (95% CI)	*P*	HR (95% CI)	*P*
ECOG PS	1.525(1.093–2.130)	0.013	1.901(1.252–2.887)	0.002
Brain metastases	2.209(1.301–3.751)	0.003	–	–
GPS	1.606(1.217–2.120)	<0.001	–	–
PD vs PR/SD	7.304(4.373–12.200)	<0.001	4.169(2.297–7.569)	<0.001
Therapeutic regimen				
Single drug	Ref.	Ref.	Ref.	Ref.
Combined chemo	0.799(0.365–1.7034)	0.545	0.154(0.021–1.118)	0.064
Combined immune	0.258(0.125–0.531)	<0.001	0.436(0.157–1.213)	0.111
Combined target	0.396(0.187–0.836)	0.015	0.239(0.070–0.816)	0.022
ΔL1SMI per 90 days	0.959(0.924–0.995)	0.025	0.898(0.854–0.945)	<0.001

(n=122).

PFS, progression-free survival; OS, overall survival; HR, hazard ratio; CI, confidence interval; ECOG PS, Eastern Cooperative Oncology Group performance status; GPS, Glasgow Prognostic Score; PD, progressive disease; PR, partial response; SD, stable disease; SMI, skeletal muscle index at the first lumbar vertebra level; VAT, visceral adipose tissue at the first lumbar vertebra level; SAT, subcutaneous adipose tissue at the first lumbar vertebra level.

Above all, patients can benefit from the newly-built nomograms and changes in L1SMI before starting treatment can detect poor outcomes.

## Discussion

The present study focused on the prognosis prediction for lung cancer patients treated with anlotinib or apatinib based on the construction of nomograms. The clinical variables contained in the establishment consisted of ECOG PS, brain metastases, GPS, PD *vs* PR/SD, therapeutic regimen, and ΔL1SMI per 90 days for PFS and ECOG PS, PD *vs* PR/SD, therapeutic regimen, and ΔL1SMI per 90 days for OS. Here, for the first time, we demonstrate that dynamic skeletal muscle change before the outset of the treatment can be used for prognosis prediction. In the setting of an advanced state with limited treatment options, screening for patients who may benefit from antiangiogenic therapy is important.

In this study, body composition measured at the L1 level was considered representative of the whole body. Given that a majority of studies have investigated CT-derived muscle mass at the L3 level to predict clinical outcomes in patients with cancer, some authors have considered the L1 as suitable alternatives for L3 in lung cancer (8, 24, 26, 27). Wei et al. concluded that single slice adipose tissue areas at different levels in the abdominal region (L4-5, 5 cm above and below) correlated well with total body adipose tissue (22). We confirmed a strong association for body composition at L1 and L3 in our patients (data not shown). Therefore, body composition analysis at the L1 level is feasible and reliable in lung cancer.

Weight loss, the most apparent feature of cachexia, has been proven to be an important poor prognostic factor in NSCLC independent of other variables (28). Weight loss >5% over the past 6 months has been widely regarded as a sign of entering the cachexia period. In our study, weight loss failed to be included in the nomograms by LASSO Cox regression analysis, whereas skeletal muscle change was identified as a prognostic factor in the final model. Low SMI at baseline or dynamic skeletal muscle alteration during treatment, as a determinant of survival, has got attention in previous studies (29–31). Low SMI presenting at baseline failed to be a prognostic factor in our patients, whereas skeletal muscle change before the initiation of the treatment significantly impaired prognosis independent of other well-established clinical prognostic indicators. This finding contributes to earlier detection of poor outcome in lung cancer patients, compared to only focusing on body composition alterations during treatment. As for body composition alterations during treatment, it was too late to ameliorate the situation.

The underlying mechanism behind how muscle loss leads to increased risk of mortality and disease progression is very complex. Muscle loss, which is a result of an imbalance between the pathways of synthesis and degradation of muscle proteins, has been associated with several biological mechanisms including systemic inflammation (32), ubiquitin proteasome pathway (33), autophagy (34, 35) and so on. Previous studies speculated that proinflammatory cytokines, including TNF-α, IL-1β, IL-6, and IL-8 may play important roles in the development of NSCLC (36–38). These factors may explain the relatively strong correlation between muscle loss and mortality. Although cancer cachexia cannot be treated with nutritional therapy alone, optimal nutritional care is recommended as a cornerstone of multimodal therapy (39). Getting enough dietary protein is a prerequisite for the maintenance or gain of skeletal muscle mass (40), whereas, the muscle wasting associated with cancer is troublesome and cannot be completely reversed with enhanced nutritional support (1). Multidrug, such as anamorelin hydrochloride (41), MABp1 (42), enobosarm (43), and several combinations were tested in the phase 3 randomized controlled trials. In the clinical studies, body weight, lean body mass, symptoms, physical functions, and prognosis have been regarded as ideal endpoints (44).

Unlike skeletal muscle, to date, there has been little agreement about the precise role of visceral and subcutaneous adipose tissue in predicting survival. Nattenmüller et al. observed that all adipose tissue compartments increased in 200 lung cancer patients during first-line chemotherapy, and the increase in SAT was associated with poor survival (45). Murphy et al. investigated the last 500 days of life in 108 lung cancer patients receiving palliative care; their findings supported the point that the extensive loss of adipose tissue is a key feature of cancer cachexia, and they observed that the loss of adipose tissue occurs at approximately 7–8 months before death and is associated with a two-fold shorter survival (46). In our Kaplan–Meier analysis for PFS and OS, loss of visceral and subcutaneous adipose tissue per 90 days before treatment was both significantly associated with poor outcome.

The combination of CRP and albumin into a score (0, 1, 2), termed as the GPS, is a widely accepted index to characterize systemic inflammation and is associated with the prognosis in advanced cancer disease (47, 48). As would be expected, significant differences in the risk of disease progression and death were observed for different values of the GPS in our research.

The combination of immunotherapy (49), chemotherapy (50, 51), or EGFR TKI (50) with antiangiogenic therapy in previous researches showed promising antitumor activity in pre-treated NSCLC. In our nomograms, therapy regimen also played an important role in influencing the prognosis, and combination therapy showed better prognosis than single drug. In the clinic, the strategy chosen for patients should not only follow the guidance but also take cost, potential survival benefit, and drug accessibility into consideration. In the real world, although the combination regimen varied, the combination of immunotherapy, chemotherapy, or EGFR TKI with antiangiogenic therapy has become more commonplace in recent years. Further large-scale studies are needed define the relationship between combination therapy and efficacy. Moreover, nomograms developed include other clinical features, such as ECOG PS, clinical response and brain metastases, which have been generally accepted as important factors in predicting prognosis. Here, we will not discuss them anymore.

We are aware of several limitations in our study. First, its retrospective single-center study design and small sample size limit the generalization of the results. Although 1,000 bootstrap re-samplings were performed to validate this model, external validation of cohorts from other centers was unavailable in our study. Despite the small sample size, the effects of body composition alteration on survival were striking when other well-established prognostic factors such as ECOG PS, therapeutic regimen, and clinical response were taken into account. Second, a possible selection bias may have occurred due to the exclusion of patients without available CT scans (21 patients). Third, the study population is strongly heterogeneous because of the different treatment regimens used. Another limitation was that body composition at L1 has not yet been validated on a large scale for predicting the prognosis of cancer patients and needs more reliable studies for support. Therefore, multicenter prospective randomized clinical trials with large sample sizes are needed to confirm our results.

In conclusion, nomograms were developed from clinical features and nutritional indicators to predict the probability of achieving 3- and 4-month PFS and 7- and 8-month OS with antiangiogenic therapy for advanced lung cancer. These nomograms may be useful to improve the management of advanced lung cancer in clinical work. Dynamic changes in body composition before the initiation of treatment contributed to early detection of poor outcome.

## Data Availability Statement

The raw data supporting the conclusions of this article will be made available by the authors, without undue reservation.

## Ethics Statement

The studies involving human participants were reviewed and approved by the ethical review board committee of Sir Run Run Shaw Hospital. The patients/participants provided their written informed consent to participate in this study. Written informed consent was obtained from the individual(s) for the publication of any potentially identifiable images or data included in this article.

## Author Contributions

Conceptualization: QP and HZ. Data collection: HoL, YF, and DL. Formal analysis: HZ and HoL. Funding acquisition: QP and HP. Investigation methodology: QP and HaL. Project administration: WH and HP. Resource and software: WZ and ZN. Supervision, validation, visualization: HH and JS. Roles/writing—original draft: HZ. Writing—review and editing: QP. All authors contributed to the article and approved the submitted version.

## Funding

This work was supported by Zhejiang medical and health science and technology program (No.2018ZD029).

## Conflict of Interest

The authors declare that the research was conducted in the absence of any commercial or financial relationships that could be construed as a potential conflict of interest.
